# The determinants of financial inclusion from the households’ perspectives in Vietnam

**DOI:** 10.1371/journal.pone.0291020

**Published:** 2023-09-01

**Authors:** Ha Son Nguyen, Chi Minh Ho, Anh The Vo, Duc Hong Vo

**Affiliations:** CBER–Research Centre in Business, Economics & Resources Ho Chi Minh City Open University Vietnam, Ho Chi Minh City, Vietnam; Shandong University, CHINA

## Abstract

Various studies have been conducted to measure financial inclusion at the country level. However, measuring financial inclusion at the household level has largely been neglected in the existing literature, particularly for emerging markets such as Vietnam. This study constructs an index of financial inclusion at the household level using the Vietnam Household Living Standard Surveys (VHLSS) in 2014, 2016, and 2018. We also identify the determinants of financial inclusion from the perspective of Vietnamese households. Our study also utilizes an ordered logit model to examine the effects of the determinants on each level of financial inclusion. Our empirical results reveal three key determinants, including (i) total income per household, (ii) relative income representing the difference between the average income of the province that the household currently lives in and the total income of this household, and (iii) the distance from the household to the nearest bank branch, are crucial factors driving the financial inclusion. While the total income per household positively enhances financial inclusion, relative income appears to reduce the degree of financial inclusion. Besides, distance to the nearest bank branch poses another challenge in achieving the financial inclusion goals in Vietnam in the future.

## 1. Introduction

Over the past decades, the literature has highlighted the multiple roles of financial inclusion, such as decreasing poverty, achieving inclusive growth, maintaining the inclusion of authorization policies, and strengthening the financial sector’s sustainability. The access of low-income households’ small and middle-sized companies to financial services would considerably increase their propensity to engage in future purchases, as would the availability of banking services [1,2]. Furthermore, Li [3] explains that the ease of financial market involvement has various positive effects on the well-being of disadvantaged family members. Moreover, by promoting digitalized payments and transactions, financial inclusion can efficiently improve the allocation of financial resources for insufficient government programs and reduce the cost of waiting, travelling, and other expenses. It is also acknowledged that financial inclusion can boost banking institutions with low-cost and steady deposits, which constitute a solid foundation for lending operations. Additionally, low-income savers and borrowers can sustain their spending habits by maintaining deposits and taking loans.

In addition, financial inclusion reduces poverty by promoting financial system activities, including investments and savings. Investments and savings, in general, are vital instruments that allow all sectors of society to engage in the official financial system. Increasing the number of persons participating in unofficial economic systems also contributes to developing more efficient monetary policies. Therefore, enhanced financial inclusion, a solid strategy, and the right political direction can reduce poverty and promote economic growth. Regarding the advantages of financial inclusion for enterprises and individuals, governments worldwide have established various programs to promote financial inclusion.

Financial inclusion has been widely regarded as an essential tool for promoting economic growth and reducing poverty in Vietnam over the past decade. After more than three decades since the "Doi Moi" economic reform in 1986, Vietnam has transitioned from one of the poorest countries to a lower middle-income country, thanks to the Vietnamese government’s various economic policies. However, sustainable economic growth and income distribution are among the most controversial issues that need to be addressed. Recently, the effects of financial inclusion on sustainable economic growth and development in Vietnam have attracted the attention of various researchers. Among efforts examining the growth effect of financial inclusion in Vietnam [4], empirical studies focus on characteristics of financial inclusion within the Vietnamese context [5,6]. Particularly, Tran et al. [7] found that financial inclusion significantly reduces multidimensional poverty in Vietnam. Besides, Vo et al. [8] suggested that corporate responsibility and bank performance matter for financial inclusion in Vietnam. However, identifying the determinants of financial inclusion, especially at the household level in Vietnam, has largely been neglected. The contributions of this study to the existing literature on financial inclusion are twofold. First, we identify key determinants of financial inclusion from the perspective of Vietnamese households, mainly focusing on the income level, the relative income at the provincial level, and the distance to the closest financial hub. Second, this study examines the role of financial inclusion in reducing income inequality in Vietnam.

In the current study, we use data from the Vietnam Household Living Standard Surveys (VHLSSs) to identify the determinants of financial inclusion at the household level in Vietnam. First, using the approach adopted in [5], we estimate the financial inclusion degree for each Vietnamese household. The Vietnam Household Living Standard Surveys (VHLSSs), conducted by Vietnam’s General Statistics Office, have been conducted biannually. The VHLSS 2018 is the latest survey when this analysis was conducted. As such, we have used three surveys in 2014, 2016, and 2018 have been used in our analysis to examine the changes in financial inclusion across Vietnamese households during this period. Our empirical analysis begins with the ordinary least squares (OLS) estimation. Specifically, financial inclusion is assigned as a categorical variable. As such, we then employ the ordered logit model (OLM) to estimate the marginal effects of household characteristics on financial inclusion.

Following this introduction, the remainder of the paper is structured as follows. Section 2 reviews relevant literature on the topic. Data and research methodology are discussed in section 3. Section 4 presents and discusses the empirical findings from this paper, followed by the conclusions in section 5 of the paper.

## 2. Literature review

### 2.1. Financial inclusion and its measurements

Initially, the term inclusive growth has been emphasized by numerous governments and development organizations, including the Organization for Economic Cooperation and Development (OECD), International Monetary Fund (IMF), the United Nations (UN), and many others [9–11]. Inclusive growth refers to comprehensive growth in which all individuals receive fairer benefits and opportunities. Many reports have examined the inclusive growth of developed nations [12–14]. While inclusive growth or inclusive green growth is a broad concept of inclusiveness in economics, inclusive finance or financial inclusion particularly focuses on the growth effects of finance [15]. Over the last few years, countries have supported financial inclusion by developing financial systems that provide access to every user in the economy. Literature has identified the multiple roles of financial inclusion, such as reducing poverty, achieving inclusive economic growth, assuring government policies’ extensiveness, or supporting the financial sector. More than 60 nations have considered financial inclusion one of the main goals to support their economic growth and development [16].

Financial inclusion and methods of estimating financial inclusion have been widely discussed in previous studies. According to [17,18], financial inclusion provides a mechanism that ensures participants can easily access, use, and benefit from the official financial system. These authors introduced the index of financial inclusion (IFI) as a multidimensional indicator which combines various banking sector indicators and captures them into one number ranging from 0 to 1, with 0 denoting complete financial exclusion and one denoting complete financial inclusion in a particular country. Many scholars use this approach to measure a degree of financial inclusion because it covers three main dimensions: (i) ease of access, (ii) availability, and (iii) usage. These three main dimensions are selected based on another famous study by [19]: the global findex database, which will be briefly reviewed later.

One of the most widely used notations of financial inclusion comes from the study of [19], which constructed a set of indicators to measure financial inclusion across nations: the Global Findex Database. This database is collected from over 148 countries worldwide, including low-income, middle-income, and high-income countries. The Global Findex Database focuses on financial services usage, not access to financial services (commonly known as financial services penetration). Unlike financial services penetration, which is heavily affected by the supply-side (i.e., financial service providers), financial services usage is affected by both the supply-side and demand-side (i.e., financial service users). In this database, they divide the collected indicators into three sub-groups: (i) indicators of account penetration, (ii) indicators of saving, and (iii) indicators of borrowing. This study contributes to the literature by filling various gaps, such as the need for systematic financial inclusion data across countries and the incomplete split of the aggregated financial inclusion data across categories. Up to the present time, this project is still updated and provided every three years, with the last version being published in 2021. Partly motivated by this study, many scholars propose new methods to capture the multidimensional characteristic of financial inclusion. [20] propose new methods to measure the level of financial inclusion mathematically. Their paper points out two critical weaknesses in the current method of calculating the multidimensional financial index. Firstly, existing financial inclusion index calculations are heavily based on supply-side data (commonly measured by the number of loans or accounts of users). However, these measures sometimes are inaccurate since one person can easily have more than a bank account or loan amount. Secondly, the assignment of weights for each dimensional indicator of financial inclusion: Access, Barriers, and Usage is mainly based on subjective evidence and a lack of mathematical concepts. They propose another unique way to calculate the financial inclusion index to build a better one responsive to all the above problems. Their improved financial index uses the two-step PCA (i.e., Principal Component Analysis) to calculate each sub-dimensional index’s weight and compute the final index based on the weighted average method [17,18]. After employing this approach to calculate the financial inclusion index for 82 countries in 2011, they found that "Access" gets the highest weight among the three sub-indexes. In other words, key factors can determine the level of financial inclusion, which means that the official financial services supply (for instance: Commercial banks) plays a more critical role in determining the financial inclusion level than the number of users.

After Sarma introduces the financial inclusion index, growing literature attempts to improve the techniques of allocating the weights of each sub-indexes. [21] also, notice shortcomings of the existing approach to measuring financial inclusion. Therefore, they aim to propose their index to overcome all the mentioned shortcomings mathematically. This paper introduces two dimensions of financial inclusion versus three in [1,17,18]. These two dimensions are the capacity of financial services and the financial services usage. This approach differs mainly from other indexes since it uses the Factor Analysis approach to group sub-dimensional indexes into the most appropriate dimension. Besides, using this approach, the randomly assigned weights based on subjective choices of other studies are well-replaced by a more mathematical way of weights assignment. Similarly, to prevent subjective weighting issues, [22] employs the Coefficient of Variation method to objectively distribute the weights among sub-dimensional indexes. After calculating the indexes for 127 countries worldwide in 2011, the geographical distribution of financial inclusion has been found: Most Asian and African countries have lower financial inclusion levels than European and North-American countries.

From another perspective, [23] introduces a broad-based method, aggregating all sub-indices into the set of financial development indicators, using familiar PCA and weighted average approaches. Financial development, a more general definition, holds many similarities in definitions compared to financial inclusion [19]. Some notable indexes that can be mentioned here are indexes of financial development, financial markets, and financial institutions. While still having many limitations regarding data availability and mathematical approaches, this study sheds essential insights into various sub-factors that can affect overall financial development. Since the dimensions of financial inclusion are often unobservable, there is no direct way to measure this index quantitatively. An updated version of [20] attempts to calculate the financial inclusion index 2014 using a two-step PCA. In terms of time effects, the financial inclusion growth from 2011 to 2014 is also provided in this paper. Most countries in the list of 137 countries have seen increased financial inclusion levels over time.

In Vietnam, the literature on financial inclusion and its measurements is growing. However, studies about measuring financial inclusion using micro-level data are still limited. The main barrier is data availability. We do not have enough micro-level data to measure all the dimensions of financial inclusion. [5] create a unique IFI using Vietnamese households’ data in their study. They employ a set of Yes/No questions about the financial status of households from section 8 of the Vietnam Household Living Standards Surveys 2018 (VHLSS 2018) to calculate a unique index by province. In this paper, they find empirical evidence that the financial inclusion level of Vietnam largely depends on income distribution at the provincial level. Moreover, the crucial economic cities, especially in Vietnam’s North and South regions, tend to have higher financial inclusions than other areas. Another study from [6] tries to measure financial inclusion with household-level data, following the guidelines of the World Bank (2018). Instead of aggregating all indicators into one single index, four-dimensional indicators of financial inclusion: Loans from formal institutions, the value of this loan, having an official bank account, and having an official saving account are employed in their model individually. They provide evidence that households in provinces with better institutional quality receive official loans, open new debit or saving accounts, and have greater access to financial opportunities.

### 2.2. The empirical evidence of financial inclusion

The literature discusses the role of financial inclusion over the past decades. [24] found that the development of the financial system and the increasing degree of financial inclusiveness contribute to overall economic growth and income distribution in Asia countries. Similarly, using a dataset of 37 Asian countries, [25] provided empirical evidence to confirm that financial inclusion significantly reduces income inequality and poverty. However, this relationship depends on the country’s demographics, such as population. Another study conducted by [26] confirms the positive and crucial role of financial accessibility in reducing income inequality, especially in low-income nations. Furthermore, they also concluded that financial inclusion can moderate the effect between economic growth and income inequality.

At the country level, numerous studies [27,28] confirm the role of financial inclusion in increasing income and reducing income inequality among individuals. For example, [27] suggested that the increasing number of bank branches opening in Mexico, which is one of the important dimensions of financial inclusion, can increase the income of individuals and reduce poverty [17–19]. Similarly, utilizing the national finance survey data of over 6,000 households in China [28] highlighted the importance of enhancing financial inclusion in improving people’s living standards, reducing poverty, and reducing income inequality in the context of Chinese households. Another interesting thing in this study is that low-income households benefit more from enhancing financial inclusion progress than wealthier households.

Regarding the crucial role of financial inclusion in social-economic development, the literature presents a comprehensive understanding of financial inclusion’s characteristics, especially at the household or individual level, which is closely related to individuals’ financial behaviours. Among the first studies on financial behaviour, [29] demonstrated that given the market imperfections, income distribution could heavily affect individual investment, spending, and saving behaviours. These behaviours directly affect the use of financial services or financial inclusion [17–19,21,27].

From a microeconomics perspective, various studies reveal the determinants of accessing the formal and informal financial markets, one of the most important pillars of financial inclusion [17–19,21,27]. Particularly, [30] found that access to credit and credit demand depends heavily on geographical and regional characteristics. While people with higher education backgrounds and living in urban areas have more opportunities to access formal credit, people from rural areas and lower education backgrounds probably use the informal system. Another paper by [31] confirmed that access to credit varies differently between rural and urban areas. Another study from [32] examines the determinants of financial inclusion in China at the individual level. Similarly, [33] assessed the determinants of financial inclusion in 12 Asian countries individually. However, [32,33] only considered financial inclusion as owning a formal account which is not an adequate measurement of financial inclusion. From macroeconomics aspects, [4,5] found that institutional quality and bank performance improve financial inclusion. Limited studies have been conducted to identify determinants of financial inclusion at the household level.

Our literature review highlights the need for academic studies to investigate the determinants of financial inclusion at household or individual levels, especially in developing countries. Data availability and difficulties in constructing the micro-level financial inclusion index might be the two critical barriers preventing such research. Financial inclusion has been considered an important source to reduce poverty and inequality in developing countries [25,34–36]. As such, our study is conducted to provide additional evidence regarding the determinants of financial inclusion at the household levels in Vietnam based on the emergence of an ongoing debate about measuring financial inclusion using micro-level data and a scarcity of empirical studies on determinants of financial inclusion in a developing nation. The contributions of our study to the current literature on financial inclusion are twofold. First, we construct an index of financial inclusion at the household level in Vietnam. Second, we identify the determinants of financial inclusion at the household level in the Vietnamese context. Vietnam is an emerging nation aiming to reduce poverty and inequality by promoting financial inclusion [6,7].

## 3. Methodology

### 3.1. Econometric model–the ordered logit model and its assumptions

An econometric equation is constructed to investigate determinants of financial inclusion in Vietnam from the household perspective:

$$LFI_{i}=\beta_{0}+\beta_{1}Age_{i}+\beta_{2}Age^{2}{}_{i}+\beta_{3}Gender_{i}+\beta_{4}Marriage_{i}+\beta_{5}Employed_{i}+\beta_{6}Edu_{i}+\beta_{7}HRI_{i}+\beta_{8}Distance_{it}+\beta_{9}log(TI_{i})+\beta_{10}Location_{i}+u_{i}$$

where:

- LFI_i_ denotes the level of financial inclusion of households i
- Age denotes the age of the household head
- Gender denotes the gender of the household head (0—Male; 1—Female)
- Marriage denotes the marriage status of the household head of household i (0—Other; 1—Married)
- Employed denotes the employment status of the household head of household i (0—Unemployed; 1—Employed)
- Edu denotes the educational level of the household head of household i
- HRI denotes the relative income of the household i
- TI denotes the total income of all individuals in the household i
- Distance indicates the nearest distance from household i to the nearest bank
- Location denotes the area where the household currently lives (0 –City/urban area; 1 –Rural area)

Financial inclusion (LFI) is categorized into three levels: Low, Medium, and High. We experience an ordered dependent variable, which takes three values: 1 –a low level of financial inclusion, 2—a medium level of financial inclusion and 3—a high level of financial inclusion. Since proportional odds logistic regression is the most common approach to deal with ordinal dependent variables among various types of the ordered logistic regression model [37,38], we employ the approach of the (partial) proportional odds model in this paper.

First, let us assign the value for the financial inclusion level of Y:

$$y=\left\{ \begin{array}{l} 1\;Low\;LFI\\ 2\;Medium\;LFI\\ 3\;High\;LFI \end{array}\right.$$ (1)

To formalize the ordinal logit model explanation, we mathematically express Eq (1):

$$y_{i}=\left\{ \begin{array}{l} 1\;if\;y_{i}^{*}\leq \kappa_{1}\\ 2\;if\;\kappa_{1}\leq y_{i}^{*}\leq \kappa_{2}\\ 3\;if\;y_{i}^{*}\geq \kappa_{2} \end{array}\right.$$ (2)

where:

- *y*_*i*_ denotes the observed ordered LFI in household i
- $y_{i}^{*}$ denotes the unobserved variable (or latent variable) to determine the value of *y*_*i*_
- *κ*_1_ and *κ*_2_ denote the threshold points (or cut-off points)

The observed variable y is also the function of the latent (And unobserved) variable y*. Considering the total population, we have the estimated equation for the latent variable:

$$y_{i}^{*}=Z_{i}+\varepsilon_{i}=\sum_{k=1}^{K}\beta_{k}x_{ki}+\varepsilon_{i}$$ (3)

where:

- Z_i_ denotes the part of the ordinal logit model’s equation
- *β* denotes the parameters that need to be estimated
- *ε*_*i*_ denotes the error term of the equation

Since the error terms *ε*_*i*_ of the above equation follows the logistic distribution with mean μ = 0 and variance s = $\frac{\pi^{2}}{3}$, we estimate its probability distribution and plug it into the equation later. Hence, we can remove it from the estimated equation. Then Eq (3) can be expressed:

$$Z_{i}=\sum_{k=1}^{K}\beta_{k}x_{ki}=E(y_{i}^{*})$$ (4)

We now come to an essential assumption of proportional odds models: the proportional odds assumption. This assumption requires that every coefficient beta in Eq (4) needs to be the same. In other words, the regression lines of i equations in (4) should be parallel. This assumption is also commonly known as the parallel lines assumption [37].

Continuing with the expression, with each value of the observed y (in this case, y takes three values: 1, 2 and 3.), Eq (4) can be changed to the probability equations:

$$P(y_{i}>j)=\frac{e^{x_{i}\beta -k_{j}}}{1+e^{x_{i}\beta -k_{j}}};j=1,2,3,\ldots ,M–1$$ (5)

Eq (5) can be expressed as:

$$P(y_{i}=1)=1-\frac{e^{x_{i}\beta -k_{j}}}{1+e^{x_{i}\beta -k_{j}}}$$

$$P(y_{i}=j)=\frac{e^{x_{i}\beta -k_{j-1}}}{1+e^{x_{i}\beta -k_{j-1}}}-\frac{e^{x_{i}\beta -k_{j}}}{1+e^{x_{i}\beta -k_{j}}};j=2,3,\ldots ,M–1$$

$$P(y_{i}=M)=\frac{e^{x_{i}\beta -k_{M-1}}}{1+e^{x_{i}\beta -k_{M-1}}}$$

Since we have three values of observed dependent variables (M = 3) and two cut-off points (M-1 = 2) in this case, the above equations are shortened to these three probability equations:

$$P(y_{i}=1)=\frac{1}{1+e^{Z_{i}-k_{1}}}$$

$${P(y}_{i}=2)=\frac{1}{1+e^{Z_{i}-k_{2}}}-P(\frac{1}{1+e^{Z_{i}-k_{1}}})$$ (6)

$$P(y_{i}=3)=1+\frac{1}{1+e^{Z_{i}-k_{2}}}$$

The interpretation of the ordered logit model is not as straightforward as in the OLS method [39,40]. Moreover, Wooldridge also suggests that the regressions are expressed as exponentiated coefficients. The log-odd ratios only inform us about the relationship of each explanatory variable but not the magnitude of these differences.

Besides, the interpretations are meaningful when the empirical results are expressed as the marginal effects (adjusted predictions) as in Eq (6). With the marginal effects, we can define values for each explanatory variable in our regression model and then calculate the probability that the event will happen for a specific individual with the chosen values [41]. Hence, this paper also provides the empirical results together with the marginal effects (the mean values for continuous variables and threshold values for factor variables).

### 3.2. Data sources

This paper utilizes the Vietnam Household Living Standards Surveys (VHLSS) in 2014, 2016, and 2018. Every VHLSS survey is conducted once every two years by the General Statistics Office, under the supervision of the Ministry of Planning and Investment of Vietnam and the World Bank’s technical assistance. This survey aims to observe the socioeconomic changes based on Vietnamese households’ living conditions. In the analysis, we will mainly focus on the data in the following three sections from the VHLSS surveys, including section 4: *Income of households*, section 5: *Expenditure of households*, and section 8: *Participating in aid schemes status of households*. The summary statistic of variables will be presented in Table 1 below.

**Table 1:** The Summary of the descriptive statistics.

2018	Obs.	Mean	Std. Dev.	Min	Max
**FI LEVEL**					
1	34,437	.89	.32	0	1
2	34,437	.11	.31	0	1
3	34,437	.01	.08	0	1
**Age**	34,437	52.44	13.95	12	113
**Age**^**2**^	34,437	2,944.12	1,560.22	144	12,769
**Gender**					
Female	34,437	.23	.42	0	1
Male	34,437	.77	.42	0	1
**Marriage status**					
Other	34,437	.47	.5	0	1
Married	34,437	.53	.5	0	1
**Employed or not**					
Unemployed	34,437	.16	.36	0	1
Employed	34,437	.84	.36	0	1
**Educational level**					
Did not complete high school	34,437	.76	.43	0	1
High school	34,437	.16	.37	0	1
College/University	34,437	.08	.27	0	1
**Urban or rural area**					
Rural	34,437	.69	.46	0	1
Urban	34,437	.31	.46	0	1
**Relative income**	34,437	1	.8	.01	25.46
**Log (Total income per household)**	34,437	11.56	.87	6.21	15.85
**Nearest bank branch distance**	34,437	9.11	3.73	4.3	14.3
**2016**	**Obs**	**Mean**	**Std. Dev.**	**Min**	**Max**
**FI LEVEL**					
1	43,459	.91	.28	0	1
2	43,459	.08	.27	0	1
3	43,459	.01	.07	0	1
**Age**	43,459	51.56	13.67	14	111
**Age**^**2**^	43,459	2,845.69	1,508.17	196	12,321
**Gender**					
Female	43,459	.24	.43	0	1
Male	43,459	.76	.43	0	1
**Marriage status**					
Other	43,459	.19	.39	0	1
Married	43,459	.81	.39	0	1
**Employed or not**					
Unemployed	43,459	.16	.36	0	1
Employed	43,459	.84	.36	0	1
**Educational level**					
Did not complete high school	43,459	.77	.42	0	1
High school	43,459	.15	.36	0	1
College/University	43,459	.08	.27	0	1
**Urban or rural area**					
Rural	43,459	.69	.46	0	1
Urban	43,459	.31	.46	0	1
**Relative income**	43,459	1.01	.89	0	41.6
**Log (Total income per household)**	43,459	11.38	.87	3.91	15.74
**Nearest bank branch distance**	43,459	10.01	3.51	5.3	15.5
**2014**	**Obs**	**Mean**	**Std. Dev.**	**Min**	**Max**
**FI LEVEL**					
1	7,959	.94	.23	0	1
2	7,959	.06	.23	0	1
3	7,959	0	.06	0	1
**Age**	7,959	51.14	13.62	16	105
**Age**^**2**^	7,959	2,801.16	1,506.69	256	11,025
**Gender**					
Female	7,959	.25	.44	0	1
Male	7,959	.75	.44	0	1
**Marriage status**					
Other	7,959	.2	.4	0	1
Married	7,959	.8	.4	0	1
**Employed or not**					
Unemployed	7,959	.34	.47	0	1
Employed	7,959	.66	.47	0	1
**Educational level**					
Did not complete high school	7,959	.77	.42	0	1
High school	7,959	.16	.37	0	1
College/University	7,959	.07	.26	0	1
**Urban or rural area**					
Rural	7,959	.69	.46	0	1
Urban	7,959	.31	.46	0	1
**Relative income**	7,959	1.01	.73	0	12.72
**Log (Total income per household)**	7,959	11.28	.84	5.52	15.21
**Nearest bank branch distance**	7,959	9.87	3.18	5.3	14.7

#### Financial inclusion level

In this study, following the method of [5], we use a similar set of eight questions in Section 8 of the VHLSS datasets to construct the IFI at the household level. Besides, we also remove the last question (m8c5) because the intention of this question is unclear to us. Since households can borrow money or goods from their relatives or neighbours, the "m8c5" question cannot represent the expected financial inclusion level. The entire question descriptions of section 8 are listed in Table 2 below.

**Table 2:** Question descriptions of section 8.

Variable	Question	Type	Section in VHLSSs
m8c3a	Has your household got a bank account at this moment?	Dummy (1 = Yes)	Section 8
m8c3b	Has your household got a savings book at this moment?	Dummy (1 = Yes)	Section 8
m8c4a	Has your household used an ATM (Debit) card at this moment?	Dummy (1 = Yes)	Section 8
m8c4b	Has your household used a credit card at this moment?	Dummy (1 = Yes)	Section 8
m8c4c	Has your household got any life insurance at this moment?	Dummy (1 = Yes)	Section 8
m8c4d	Has your household got any non-life insurance at this moment?	Dummy (1 = Yes)	Section 8
m8c4e	Has your household got any stock or securities at this moment?	Dummy (1 = Yes)	Section 8
m8c5	Has your household borrowed any money or goods over the last 12 months?	Dummy (1 = Yes)	Section 8

Source: VHLSS datasets from 2014 to 2018.

The original approach to estimating the provincial financial inclusion index from Nguyen et al. (2021) is simple and effective. First, each household in a province answers all these eight questions. All scores are then summed across provinces. Finally, the total is divided by the product of the total number of households in this province and the total number of questions. This approach ensures that each provincial index ranges between zero and one (i.e., normalizing process). In this study, we construct a household-level financial inclusion index. Hence, in the first step, we aggregate the results of all the above questions (excluding the "m8c5" question) into one single financial inclusion index for each household:

$$IFI_{i,t}=m8c3a_{i,t}+m8c3b_{i,t}+m8c4a_{i,t}+m8c4b_{i,t}+m8c4c_{i,t}+m8c4d_{i,t}+m8c4e_{i,t}$$

where:

- IFI_I,t_ denotes the financial inclusion index of household i in year t
- M8c3a_i;t_; M8c3b_i;t_; M8c4a_i;t_; M8c4b_i;t_; M8c4c_i;t_; M8c4d_i;t_; M8c4e_i;t_ is the Yes/No answers to the questions of the household i in year t (Takes the value 0 or 1 only)

Next, each IFI is categorized into three levels of financial inclusion (LFI), as shown in Table 3 below.

**Table 3:** Categorizing the level of financial inclusion across provinces in Vietnam.

Financial inclusion index (LFI)	Level of financial inclusion (LFI)
From 0 to 2	Low level–Takes the value of 1
From 3 to 4	Medium level–Takes the value of 2
From 5 to 7	High level–Takes the value of 3

#### Total income of each household

In the original VHLSS data, no question directly asks regarding the household’s total income. As such, we manually calculate this income based on selected questions in section 4: *Income*. This section divides the household’s income into five groups: Part 4A - *Income from wages and salaries*, part 4B –*Income from agriculture activities*, part 4C –*Income from business*, *production*, *and other non-agriculture activities*, and part 4D –*Other revenues*. At the end of each part, they have a question that sums up all the revenue and cost, which we utilize to calculate the total income for the households in Vietnam.

#### The household’s “relative income” index

According to [3,42], the relative income (Or distance income) represents the difference between the average income of the province that the household currently lives in and the total income of this household. [42] used Vietnam’s Access to Resources Household Survey (VARHS) to calculate relative income in the original paper. Motivating by this approach, but with changes in calculating the average income of all household members at the district level, we construct the household relative income (HRI) variable using the VHLSS surveys:

$$HRI_{i,t}=\frac{{TI}_{i,t}}{{AIP}_{t}}$$

where:

- TI_i,t_ denotes the total income of all members in household i in year t
- AIPI_t_ represents the average income of the district that this household currently lives in

#### Distance to the nearest commercial bank

For the distance from the households to the nearest bank variable, as this information does not have in the questionnaires of VHLSS datasets, the data of this variable is manually gathered using the VHLSS handbook of the General Statistics Office (GSO), which is also known as “The results of the VHLSS 2018 handbook”. In section 11.21: *Access to the infrastructure by region*, the average distance from households to their nearest financial institution at the provincial level is provided. This paper uses the distance from households to the nearest official bank branch as one of the leading determinants of financial inclusion.

## 4. Results and interpretations

### 4.1. Financial inclusion level across provinces in Vietnam and its determinants

**Fig 1:**
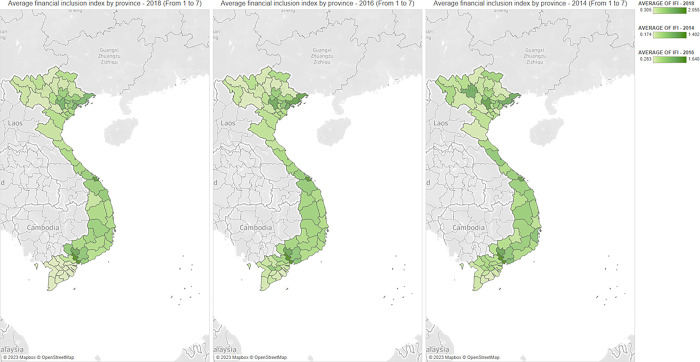
The average value of the financial inclusion index by province (From 1 to 7). Source: Authors’ visualizations using OpenStreetMap’s open data (Contains information from OpenStreetMap and OpenStreetMap Foundation, which is made available under the Open Database License).

**Fig 2:**
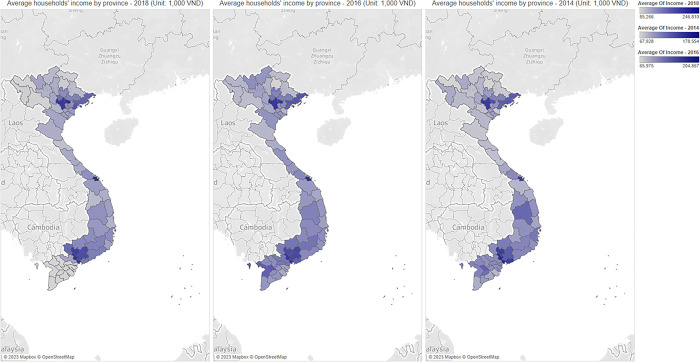
The average value of households’ income level by province (Unit: 1,000 VND). Source: Authors’ visualizations using OpenStreetMap’s open data (Contains information from OpenStreetMap and OpenStreetMap Foundation, which is made available under the Open Database License).

**Fig 3:**
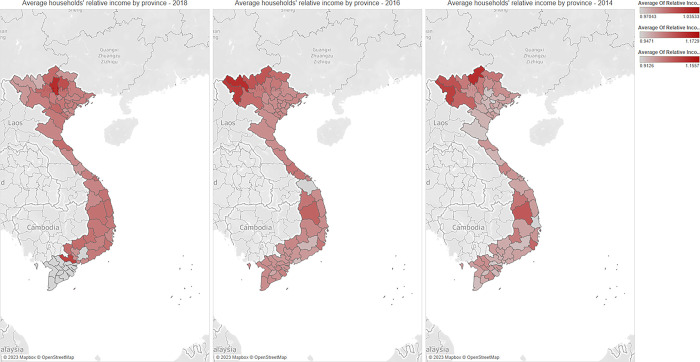
The average value of households’ relative income level by province (Unit: 1,000 VND). Source: Authors’ visualizations using OpenStreetMap’s open data (Contains information from OpenStreetMap and OpenStreetMap Foundation, which is made available under the Open Database License).

In this section, we present the average financial inclusion level across provinces in Vietnam, households’ income, and their relative income index in Figs 1–3. These figures capture the potential differences in the level of financial inclusion and its determinants, including the households’ income and relative income index across provinces, and predict the relationship among these three variables.

As presented in Figs 1 and 2, from 2014 to 2018, the distribution of financial inclusion across provinces was heavily unbalanced. In urban cities in North and South regions (such as Ha Noi and Ho Chi Minh City), financial inclusion is significantly higher than in other areas. These findings align with previous studies [5,6]. The distribution of household income levels across provinces follows the same pattern because households in central provinces tend to receive higher incomes than other provinces. These observations imply the important role of financial inclusion in increasing households’ income, especially in key economic cities and regions. Interestingly, the visualizations from Fig 3 reveal the opposite pattern regarding the households’ relative income. Our results indicate that the relative income of the central areas in the North and South regions is smaller than in non-central areas. These findings confirm the inequality in income distribution across households, which is negatively correlated with financial inclusion.

### 4.2. The effects of income, relative income, and distance to the nearest commercial banks on financial inclusion at the household levels in Vietnam using the ordinary least Square (OLS) with robust standard errors and the ordered logit model (OLM)

Generally, with discrete and ordinal dependent variables such as the level of financial inclusion, as we currently employ in this paper, the ordinary least square estimation might provide biased results because the OLS only treats the dependent variables as continuous values. As such, the (partial) proportional-odd model (Or the ordered logit model) is the most appropriate estimation approach to examine the relationship between the level of financial inclusion and other explanatory variables [39].

It is generally accepted that the linear model using the OLS estimation can still provide a reasonable estimate of the real partial effects of independent variables on the dependent variable [40]. Hence, the OLS is used in this paper as a first-step regression to capture the signs of coefficients of each regressor, using the financial inclusion index (IFI) as the dependent variable. As previously discussed, unlike the level of financial inclusion (LFI), which only takes three values from 1 to 3, the IFI takes eight values from 0 to 7, reducing possible biases from OLS regression. We can also compare the regression results from OLS and the ordered logit model to provide meaningful interpretations. Another benefit of using the OLS estimation at the first step is that we can quickly run after-regression diagnostics (Post tests) to detect possible and conspicuous problems such as heteroskedasticity and multicollinearity.

As presented in Table 4, results from the Breusch-Pagan test indicate that all OLS regression results in 2018, 2016, and 2014 suffer heteroskedasticity, which can violate one of the crucial assumptions of OLS–a requirement of the error terms to have constant variances for observed samples. White suggests that with large sample sizes, the heteroskedasticity in the OLS estimation can be corrected using robust standard errors [43] to overcome this problem.

**Table 4:** Breusch-Pagan test for heteroskedasticity issues.

	Breusch-Pagan test for heteroskedasticity		
H_0_: Constant variance	**2018**	**2016**	**2014**
chi2(1)	15,788.45	23,470.48	6,604.85
Prob > chi2	0.0000	0.0000	0.0000

*Notes*: *A significant test statistic indicates that the null hypothesis H*_*0*_ *has been rejected*.

In Table 5 below, we summarize this regression’s (exponential) coefficients and the coefficients of the OLS regression with robust standard errors, together with the expected sign of each variable based on the literature review.

**Table 5:** The summary of the estimated coefficients–the OLM versus OLS estimations.

	2018		2016		2014	
**Variables**	**OLM** **coefficient**	**OLS** **coefficient**	**OLM** **coefficient**	**OLS** **coefficient**	**OLM** **coefficient**	**OLS** **coefficient**
Age	-0.0153	-0.00102	0.0864***	0.00453***	0.0216	-0.0000395
Age^2^	0.000125	0.00000949	-0.000750***	-0.0000373***	-0.000201	0.00000357
Gender	0.000848	-0.00457	-0.271***	-0.0287***	-0.0862	-0.0220*
Marriage status	0.00139	0.00297	0.383***	0.0350***	0.0692	0.0116
Employed	-0.303***	-0.0448***	-0.303***	-0.0295***	-0.0613	-0.00369
Graduated from high school	0.00666	0.00523	0.926***	0.0802***	0.457***	0.0222*
Graduated from college/university	0.0775	0.0166*	1.717***	0.262***	0.949***	0.148***
Living in urban areas	0.840***	0.127***	0.779***	0.0768***	0.800***	0.0664***
Relative income	-0.283***	0.0315***	-0.176***	0.00100	-0.299***	0.0338**
Log (Total Income)	2.016***	0.0896***	1.012***	0.0453***	2.105***	0.0374***
Distance (Kilometer)	-0.0180**	-0.00343***	-0.0224***	-0.00144***	-0.00324	-0.00221*

Source: Author’s calculations using Stata.

The signs of the ordered logit model (OLM) and the OLS’s coefficients are the same across three regressions in 2018, 2016, and 2014, except for Age, Age^2^, Gender, and Relative income. Besides, the expected signs of four variables: Graduated from high school, Graduated from college/university, Living in urban areas, Relative income, Log (Total Income), and Distance is not different from our regression results, implying that the empirical results of these determinants are supported by previous literature. Moreover, the different signs of relative income coefficients between OLM and OLS might suggest that the OLM method yields better estimated and unbiased results than OLS because these negative relationships from the OLM results are well supported by previous studies [25,28,44]. Although there is limited literature about the relationship between employment status and financial inclusion, the negative signs across years in both OLM and OLS estimation results are also an interesting point to look at since it contradicts with the view that employed household heads tend to get better access to financial services and banking system, therefore have higher levels of financial inclusion.

#### The marginal effects interpretations of key determinants

The average marginal effects using the generalized OLM in 2014, 2016 and 2018 are presented in Table 6 below. Unlike the exponential coefficients and log-odds values, interpreting the average marginal effect is straightforward since it can provide the predicted probabilities at each value of the financial inclusion index, depending on the changes of our independent variables. As presented in Table 6, except for Age, Age^2^, Gender, and Married status, all other variables do not change the signs of the average marginal effects across years. As such, for these variables, we focus on interpreting the effects in 2018 and then compare the magnitude of these effects with the 2016 and 2014 surveys.

**Table 6:** The average marginal effects (AMEs).

	2018		2016		2014	
	**AME**	**t-stats**	**AME**	**t-stats**	**AME**	**t-stas**
**Age**						
Low LFI (LFI = 1)	0.0012	(0.0007)	-0.0056***	(0.0007)	-0.0010	(0.0013)
Medium LFI (LFI = 2)	-0.0011	(0.0006)	0.0052***	(0.0006)	0.0009	(0.0012)
High LFI (LFI = 3)	-0.0001	(0.0000)	0.0004***	(0.0001)	0.0001	(0.0001)
**Age** ^**2**^						
Low LFI (LFI = 1)	-0.0000	(0.0000)	0.0000***	(0.0000)	0.0000	(0.0000)
Medium LFI (LFI = 2)	0.0000	(0.0000)	-0.0000***	(0.0000)	-0.0000	(0.0000)
High LFI (LFI = 3)	0.0000	(0.0000)	-0.0000***	(0.0000)	-0.0000	(0.0000)
**Gender**						
Low LFI (LFI = 1)	-0.0002	(0.0038)	0.0183***	(0.0034)	0.0039	(0.0062)
Medium LFI (LFI = 2)	0.0002	(0.0036)	-0.0168***	(0.0031)	-0.0036	(0.0058)
High LFI (LFI = 3)	0.0000	(0.0003)	-0.0015***	(0.0003)	-0.0003	(0.0005)
**Marriage status**						
Low LFI (LFI = 1)	-0.0000	(0.0033)	-0.0230***	(0.0035)	-0.0030	(0.0081)
Medium LFI (LFI = 2)	0.0000	(0.0030)	0.0213***	(0.0032)	0.0028	(0.0075)
High LFI (LFI = 3)	0.0000	(0.0002)	0.0018***	(0.0003)	0.0002	(0.0006)
**Employed**						
Low LFI (LFI = 1)	0.0256***	(0.0044)	0.0209***	(0.0037)	0.0028	(0.0050)
Medium LFI (LFI = 2)	-0.0238***	(0.0041)	-0.0192***	(0.0034)	-0.0026	(0.0046)
High LFI (LFI = 3)	-0.0018***	(0.0003)	-0.0017***	(0.0003)	-0.0002	(0.0004)
**Graduated from** **high school**						
Low LFI (LFI = 1)	-0.0006	(0.0042)	-0.0642***	(0.0037)	-0.0204**	(0.0063)
Medium LFI (LFI = 2)	0.0006	(0.0039)	0.0602***	(0.0035)	0.0192**	(0.0059)
High LFI (LFI = 3)	0.0000	(0.0003)	0.0039***	(0.0003)	0.0013**	(0.0005)
**Graduated from** **college/university**						
Low LFI (LFI = 1)	-0.0061	(0.0057)	-0.1576***	(0.0063)	-0.0502***	(0.0086)
Medium LFI (LFI = 2)	0.0057	(0.0053)	0.1461***	(0.0058)	0.0468***	(0.0081)
High LFI (LFI = 3)	0.0004	(0.0004)	0.0115***	(0.0009)	0.0034***	(0.0009)
**Living in urban areas**						
Low LFI (LFI = 1)	**-0.0702** ***	(0.0036)	**-0.0525** ***	(0.0031)	-0.0363***	(0.0056)
Medium LFI (LFI = 2)	**0.0627** ***	(0.0036)	**0.0475** ***	(0.0030)	0.0340***	(0.0053)
High LFI (LFI = 3)	**0.0074** ***	(0.0008)	**0.0050** ***	(0.0007)	0.0023***	(0.0005)
**Relative income**						
Low LFI (LFI = 1)	0.0235***	(0.0023)	**0.0119** ***	(0.0018)	0.0133***	(0.0038)
Medium LFI (LFI = 2)	-0.0219***	(0.0021)	**-0.0115** ***	(0.0017)	-0.0124***	(0.0036)
High LFI (LFI = 3)	-0.0016***	(0.0002)	**-0.0004**	(0.0002)	-0.0010**	(0.0003)
**Log(Total income)**						
Low LFI (LFI = 1)	**-0.1611** ***	(0.0039)	-0.0660***	(0.0028)	-0.0941***	(0.0067)
Medium LFI (LFI = 2)	**0.1468** ***	(0.0037)	0.0607***	(0.0026)	0.0873***	(0.0063)
High LFI (LFI = 3)	**0.0143** ***	(0.0011)	0.0053***	(0.0004)	0.0068***	(0.0014)
**Distance**						
Low LFI (LFI = 1)	**0.0015** ***	(0.0005)	**0.0015** ***	(0.0004)	0.0001	(0.0008)
Medium LFI (LFI = 2)	**-0.0017** ***	(0.0005)	**-0.0016** ***	(0.0004)	-0.0001	(0.0007)
High LFI (LFI = 3)	**0.0002**	(0.0001)	**0.0001**	(0.0001)	-0.0000	(0.0001)
*N*	34437		43459		7959	

Notes: Standard errors in parentheses

^*^ *p < 0*.*05*

^**^ *p < 0*.*01*

^***^ *p < 0*.*001*. *Variables that are non-restricted to the parallel-lines assumption are emboldened*.

First, consider the total income’s average marginal effects (AMEs) as an example. The AMEs of the log(Total income) at LFI = 1 (-0.1611), LFI = 2 (0.1468), and LFI = 3 (0.0143) in 2018 imply that when other variables being held constant, on average, if the income of one particular household increases by one unit, the probability that the financial inclusion level (LFI) of this household is approximately 16.11% less likely to be in the group of low LFI, 14.68% more likely to be in the group of average LFI and 1.43% more likely to be in the group of high LFI. The same signs of AMEs were also found in 2016 and 2014. This finding is also consistent with previous literature about the positive relationship between financial inclusion and income in general [22,25,28,31,45].

Second, for the relative income variables (HRI), the AMEs of the HRI at LFI = 1 (0.0235), LFI = 2 (-0.0219), and LFI = 3 (-0.0016) in 2018 imply that if the relative income of one particular household increases by one unit, the probability that the level of financial inclusion (LFI) of this household is approximately 2.35% more likely to be in the group of low LFI, 2.19% less likely to be in the group of average LFI and 0.16% less likely to be in the group of high LFI. This result is in line with past studies [25,28,44].

Third, the AMEs of the distance from households to the nearest commercial bank are mixed across levels of financial inclusion. We find that if the distance to the nearest commercial bank of one household increases by one unit, the probability that the level of financial inclusion (LFI) of this household is approximately 1.5% more likely to be in the group of low LFI, 1.7% less likely to be in the group of average LFI and 0.02% more likely to be in the group of high LFI. Although the distance from the financial institution is one of the main barriers that lower the financial inclusion level [17–20,23], the effect of distance on the high level of financial inclusion is relatively small compared to other determinants.

The effects of other factors like Age, Age^2^, Gender, Married, Employed, and Education on financial inclusion have now been considered. The effect of Age and Age^2^ is noticeably small in affecting the financial inclusion level, which is different from the findings of [31]. The effect of gender seems to vary across the years, with only the result in 2016 being significant. The conclusion here is that being male acts as the factor that enhances financial inclusion only when the financial inclusion level of the household is low. Interestingly, the results also show that married and high-educated household heads positively affect financial inclusion levels only at medium and high financial inclusion levels. The distance to the nearest commercial banks also affects financial inclusion. Households living in urban areas have 7.02% less likely to be in the low financial inclusion level, 6.27% more likely to be in the average level, and 0.75% more likely to be in the high financial inclusion level.

## 5. Conclusions and implications

Over the past decades, a significant body of literature has emphasized the role of financial inclusion in improving individuals’ income, reducing poverty, and ameliorating income inequality. Nevertheless, with the growing global literature, limited studies have been conducted in Vietnam. Previous studies focus on examining the characteristics of financial inclusion and its effect on economic growth in Vietnam. Unlike previous studies, this study is conducted to identify the key determinants affecting financial inclusion degrees in the Vietnamese context using micro-level datasets. Our study uses Vietnam’s Household Living Standard Survey (VHLSS) 2018, 2016, and 2014 datasets. Key findings are summarised as follows. *First*, when households have equipped with a low financial inclusion level, increased income is associated with a reduced financial inclusion level. However, increased income improves the financial inclusion level for households when they belong to a medium or high financial inclusion level. These findings imply that a household’s initial financial inclusion level is important. *Second*, relative income positively impacts financial inclusion if this household experiences a low financial inclusion level. However, the effect is reversed when the households have a medium or high level of financial inclusion. *Third*, the location of households and the distance from their houses to the nearest bank is crucial in improving financial inclusion, especially when the households experience a medium financial inclusion level.

Policy implications have emerged based on the findings of this study. By implementing policies supporting financial inclusion, policymakers help increase individuals’ income and reduce income inequality across households in Vietnam. Important aspects of financial inclusion from both the demand and supply sides can be improved. Policies should encourage commercial banks in Vietnam to ease the new credit/debit account opening procedure, increase the number of ATMs, and motivate people to participate in insurance programs, especially in rural areas and small cities. Besides, policies motivating individuals to attend higher education, especially at the college/university level, can be considered one of the practical aspects that can improve the overall financial inclusion level. Commercial banks are encouraged to open new offices or branches to reduce the distance between households and financial institutions, especially in rural areas and less-developed provinces.

In addition, encouraging the use of formal credit is important to support financial inclusion in China [32]. Besides, supporting education helps enhance financial inclusion [33]. Encouraging women to participate in the financial system and improving trade freedom and quality of public services help support financial inclusion [46], or improving institutional quality, bank performance, and corporate social responsibility leads to better financial inclusion [4,8].

Our study exhibits limitations. Particularly, we only focus on cross-sectional analysis in the current study. We observe that available data is significantly lower when we combine data from the different waves of the VHLSS surveys. It is because respondents in previous surveys are not required to attend the surveys in the future. As such, results from these cross-sectional data are presented separately. Future studies may need to consider the time-varying effects of fundamental determinants on financial inclusion using panel data regression methodologies.
